# Attitudes and perspectives of older adults on technologies for assessing frailty in home settings: a focus group study

**DOI:** 10.1186/s12877-021-02252-4

**Published:** 2021-05-08

**Authors:** Chao Bian, Bing Ye, Anna Hoonakker, Alex Mihailidis

**Affiliations:** 1 Institute of Biomedical Engineering, University of Toronto, Toronto, Canada; 2 Toronto Rehabilitation Institute, University Health Network, Toronto, Canada; 3 Department of Occupational Science and Occupational Therapy, University of Toronto, Toronto, Canada; 4 Department of Psychology, Utrecht University, Utrecht, The Netherlands

**Keywords:** Frailty, Assessment, Technology, Sensors, In-Home Health Monitoring, Attitudes, Focus Group

## Abstract

**Background:**

The rapid development of technology such as sensors and artificial intelligence in recent years enables monitoring frailty criteria to assess frailty early and accurately from a remote location such as a home. However, research shows technologies being abandoned or rejected by users due to a lack of compatibility and consumer involvement in selecting their assistive technology devices. This study aims to understand older adults’ perceptions and preferences of technologies that can potentially assess frailty at home.

**Methods:**

This study collected qualitative data through focus group meetings with 15 participants ages 65 and older. Researchers asked participants questions to achieve the goal of understanding their attitudes on the technologies. These questions include (1) the concerns or barriers of installing and using the presented technology in daily life at home, (2) the reasons participants like or dislike a particular technology, (3) what makes a specific technology more acceptable, and (4) participants’ preferences in choosing technologies. Data were transcribed, coded and categorized, and finally synthesized to understand the attitudes towards presented technologies.

**Results:**

Three focus group sessions were conducted with five participants in each session. In the findings, the attitudes and perspectives of participants on the technologies for assessing frailty were categorized into four themes: (A) general attitude towards using the technologies, (B) conditions for accepting certain technologies, (C) existing living habits or patterns related to using the technologies, and (D) constructive suggestions related to the technologies.

**Conclusions:**

Participants generally had positive attitudes towards allowing the technologies to be installed and used at their homes. They would accept some technologies if used under certain conditions. However, questions and concerns remain, such as concerns about privacy, functionality, and aesthetics. The study also found that older adults’ living habits or patterns could affect the design and use of technology. Lastly, many valuable suggestions have been made by participants. These perspectives and insights can help improve the design and adoption of home-based frailty assessment technologies among older adults.

## Introduction

### Background

Frailty is a clinical condition that plays a role in the aging process. It is defined as a “medical syndrome with multiple causes and contributors that is characterized by diminished strength, endurance, and reduced physiologic function that increases an individual’s vulnerability for developing increased dependency and/or death’’ [1]. Based on 21 cohorts involving 61,500 participants, on average, 10.7 % of community-dwelling older adults are frail, and another 41.6 % are pre-frail [2]. It is crucial to understand frailty because frail older patients may encounter greater complexity in treatment choices, care planning, and costs of care [3]. Identification and early detection of frailty will enable personalized care by selecting an appropriate treatment plan, which could reverse frailty [4–6]. Current clinical assessments have limitations such as relying on well-trained clinicians to interpret results or being too complex to be administered [3]. The technology could be the solution to assess frailty clinically and remotely as healthcare resources become limited to the aging population.

A systematic review on assessing frailty using technologies in home settings found an increasing number of research trials in recent years. These studies used sensors and artificial intelligence to monitor frailty criteria, such as walking speed, muscle strength, physical activities, to assess frailty early and accurately from a remote location such as a home [7]. However, research shows that technologies are abandoned or rejected by users due to a lack of compatibility and consumer involvement in selecting their assistive technology devices [8, 9]. Therefore, it is necessary to know and understand older adults’ needs and requirements when designing a useful and usable technology-based frailty assessment toolkit. The design of this toolkit can take a user-centered approach. A user-centered approach incorporates user requirements, user goals, and user tasks into the design process [10, 11]. The success of the design depends on older adults being able to use the proposed technology. As such, it is necessary to research and incorporate the users in the process of designing a new solution.

It starts with gaining insights into the user’s preferences, needs, wishes, and attitudes regarding using technology to monitor health. The insights will help guide the design of a technology toolkit for assessing and monitoring frailty at home. Involving the targeted audience in the design process before creating the toolkit will allow us to select technologies for the frailty toolkit which are appreciated and more likely to be successfully adopted by the target population. Focus group discussions are a frequently used method to obtain knowledge, perspectives, and attitudes of people about issues [9]. Focus groups can provide valuable information for developing new technologies [12]. In health services research, focus groups’ primary goal is to elicit information to answer research questions [13].

Multiple studies researching the use of health technologies use focus groups to reveal usability issues within these technologies [14–18]. Several focus group studies have provided significant insights into user’s perceptions about health monitoring [12, 19, 20]. An example of a focus group study in Sweden explored the perceptions of patients with Parkinson’s disease regarding the use of wearable technology for disease monitoring and management [21]. Another example of a focus group study involving older adults and smart home technologies shows this method is valuable to gain insights for improving these technologies. This study resulted in several recommendations for the visualizations used in health monitors [22].

### Research Aim

This study aims to understand older adults’ perceptions and preferences on technologies that can be potentially used to measure frailty criteria in home settings.

## Methods

### Design

This study is a qualitative study in which data were collected through focus group interviews. We conducted three focus group sessions with older adults to evaluate their perceptions and preferences on multiple different types of technologies in the context of frailty and assessing frailty using technologies in home settings. Ethical approval was obtained from the Research Ethics Board at the University of Toronto.

### Recruitment and Participants

The inclusion criteria include:

- Be aged at least 65 years and older.
- Be fluent in English.
- Cognitively able to participate in the study for up to 90 min.
- Cognitively able to provide informed consent as determined by the study protocol..

We intended to include both participants who have or have not experienced any physical decline while aging. Therefore, participants were asked to self-report their physical health using a 5-point Likert scale before attending the focus group [23, 24]. Participants were excluded from this study if they were not fluent in English (verbal, reading, and writing) or deemed unsuitable or unable to provide informed consent.

Studies on focus group size typically recommended group sizes between 6 and 12 participants [9, 13]; Quine & Cameron [25] found the ideal group size of a focus group with older adults is between 5 and 6 participants. They described groups with four participants may have a risk of being less dynamic. However, with more than six members in a group, it can be challenging to ensure audibility and eye contact. Therefore, three focus group sessions were held with five participants in each group session. The first two sessions were in-person before the COVID-19 pandemic at the Intelligent Assistive Technology and Systems lab (IATSL) at the University of Toronto. The third session was held online using Microsoft Teams due to the pandemic. The three focus group sessions occurred in January, February, and August in the year of 2020.

 Participants were recruited from AGE-WELL NCE (Canada’s largest network in technology and aging) and local hospitals (University Health Network) in Toronto, Canada, by sending group emails and posting paper flyers.

A researcher (BY) reviewed the study with potential participants who have initiated the contact and expressed their interest. The researcher explained the study, consent procedures and answered questions. Participants were also asked to self-report their physical ability to determine eligibility. To assess cognitive competency, the researcher asked the participants questions about the consent form content to ensure they fully understand the study details. The researcher reviewed the consent form with eligible participants in person or over the phone and obtained signed consent forms before the data collection started.

### Data Collection

All focus group sessions were audiotaped. Each focus group session took 90 min with a short break in the middle of the session. Two researchers were present in all three sessions. One researcher (BY) took field notes while the other researcher (CB) asked questions and facilitated the discussion between study participants.

During the focus group, the researcher first briefly introduced the study protocol while encouraging the participants to actively participate in the discussions by stressing that there was no right or wrong answer. The researcher then introduced the concept of frailty, and the components of the frailty phenotypes defined in a clinical frailty scale, the Fried’s frailty index [26]. The researcher subsequently introduced more clinical manifestations of frailty described in the “cycle of frailty” proposed by Fried’s et al. [27] and other behavior precursors related to frailty [28]. In summary, the frailty criteria introduced included grip strength, weight loss, food intake, exhaustion, physical activities, immobilization, and life space. Lastly, the researcher presented ten different types of technologies (Table 1) and explained how to use them to assess and monitor frailty at home based on the introduced frailty criteria. These technologies were chosen because they were readily available and commonly used to detect the frailty criteria [7]. The researcher showed the technologies one by one to the participants with immediate discussion after presenting each technology before moving to the next. The discussion included questions focused on understanding if participants would feel comfortable having their health measured by the technology. Questions from different perspectives were asked to understand their attitudes toward the usefulness of the technologies. These perspectives include (1) the concerns or barriers of installing and using the presented technology in daily life at home, (2) the reasons participants like or dislike a particular technology, (3) what makes a specific technology more acceptable, and (4) participants’ preferences in choosing technologies.

**Table 1:** Technologies and the corresponding frailty criteria discussed in focus groups

Technology	Frailty Criteria to be Detected	Where to Install
Smartwatch	Physical Activity, Immobilization	Body worn
Chair and Bed Sensor	Physical Activity, Immobilization	Chair and bed
Motion Sensor	Physical Activity, Immobilization	On the wall of each room
Standard Camera	Physical Activity, Immobilization	On the wall of each room
Depth Camera	Physical Activity, Immobilization	One the wall of each room
Door Sensor	Life Space	On the edge of a door and door frame
Hand Dynamometer	Grip Strength	Portable
Fridge Door Sensor	Food Intake	On the door of a fridge
Smart Speaker	Food Intake	On a flat surface, e.g., a countertop
Smart Speaker	Exhaustion	On a flat surface, e.g., a countertop
Bathroom Scale	Weight	On the ground

### Data analysis

Qualitative data from the recordings were transcribed verbatim. The transcripts were entered and analyzed with an inductive thematic analysis [29, 30] in NVivo – QSR International software (for Mac, Release 1.3). This analysis started with open coding, writing notes, and headings in the original transcript. These headings were collected onto coding sheets, and categories or themes were generated. After the open coding, the list of categories grouped under higher-order headings. Lastly, categories were abstracted in terms of formulating a general description of the research topic. Subcategories with similar themes were grouped as categories, and categories were grouped as main categories [29]. Two members (BY and CB) from the research group performed the data analysis. Both members achieved the consensus in coding and categorization through discussion. The validity of interpretations was then discussed and agreed upon with other members of the research team.

## Results

A total of 15 older adults age 65 and older were recruited in the study. Three focus group sessions were conducted with five participants in each session. There were nine female and six male participants in total. Table 2 illustrates the demographic information of participants in three focus groups.

**Table 2:** Demographics of participants in focus groups

	ID	Age	Gender	Education level	Total years of education
**Focus Group 1**	1	84	Female	High school	12
	2	70	Male	High school	12
	3	85	Female	Masters	20
	4	66	Male	University	17
	5	74	Male	University	16
	**Mean (SD)**	**75.80 (8.44)**			**15.40 (3.44)**
**Focus Group 2**	6	65	Female	University	17
	7	69	Female	High school	9
	8	67	Female	Masters	19
	9	71	Female	College	16.5
	10	68	Female	College	17
	**Mean (SD)**	**68 (2.24)**			**15.70 (3.87)**
**Focus Group 3**	11	69	Male	College	15
	12	73	Female	Masters	18
	13	66	Male	College	16
	14	74	Male	Masters	18
	15	69	Female	College	15
	**Mean (SD)**	**70.20 (3.27)**			**16.40 (1.52)**

In the findings, the attitudes and perspectives of participants on the technologies that could be potentially used for assessing and monitoring frailty were presented in four predominant areas: (A) general attitude towards using the technology, (B) conditions for accepting certain technologies, (C) existing living habits or patterns related to use of the technologies, (D) constructive suggestions related to the technologies. Tables 3, 4, 5, and 6 provide an overview of the results for each area.

**Table 3:** Attitudes towards technologies potentially for assessing frailty

Technology	Willingness to Try	Attitudes	Reasons for the Attitudes	Concerns
Smartwatch	Yes	Positive and Negative	Positive: • Experience • Easy to wear • Not easy to lose • Lightweight Negative: • No need because of good health • Complex to set up and use • Simply no interest • Cannot see a benefit	Poor adherence
Chair and Bed Sensor	Yes	Positive and Slightly Negative	Positive: • Easy to use • Looks like something that the user would forget that it was there • Simple device Negative: • No need because of good health	Not aesthetically pleasing, feeling guilty, Not washable, battery or plug-in power supply
Motion Sensor	Yes	Positive	• No need to wear • No need to maintain	Inaccurate data
Standard Camera	No	Negative	• Privacy invasive • No need because of good health	Privacy invasive
Depth Camera	Yes	Positive	None	None
Door Sensor	Yes	Positive and Slightly Negative	Positive - None Negative: • Useless • No need	Inaccurate data
Hand Dynamometer	Yes	Positive	• Simple to use	Poor adherence, needs a reminder
Fridge Door Sensor	Yes	Positive	None	Inaccurate data
Smart Speaker	Yes	Positive and Slightly Negative	Positive: • Experience • Being fun to talk to • Had exhaustion issue Negative: • No need because of good health • Being stupid to talk to	Interaction
Bathroom Scale	Yes	Positive and Slightly Negative	Positive: • Experience • Parents had cancer Negative: • No need because of good health	None

### A) General attitudes towards using the technology

All participants reported an overall positive attitude towards each discussed technology except the standard camera. All participants showed their interest and believed there would be significant benefits of using technologies to monitor health. Overall, participants were willing to install and test out the technologies in their homes. Table 3 shows a summary of the attitudes.

One participant could see that the technologies could track changes or progress of an individual’s health conditions at home by comparing the data collected by the technologies at different times. She also added, *“it’s usually something else behind it that causes the hip to break it in the first place,”* to show that she thought people could use the technologies to understand the underlying causes of some adverse outcomes. The technologies could potentially prevent adverse outcomes such as fractions from happening by detecting early signs. Three other participants believed the data collected and interpreted by the technologies would be useful for both older adults themselves and clinicians to understand their health better and make a more informed decision.

*I think if technology like this or you know instruments like this are going to be useful for medical practitioners, then they would be valuable, and I would use it because I think the more information you can give to your doctor, the better off here. She’s going to be when it comes to treating something that you might whether its frailty or whatever and if things like this can help improve the quality of people’s lives as we age, then I think it’s a good thing*.

Participants have also seen the technologies overall could benefit the daily living of older adults. One participant thought the technologies could identify and inform potential problems in their daily living and advised them to live healthier lives. Another benefit that the participants have seen was that the more information the technologies could collect and shared with their doctors, the better the doctor could give them advice or diagnosis. Having the technologies at home monitoring their health, participants believed it could promote aging in place with longer independence.

Eight participants across three focus groups indicated that they had experience using a smartwatch, such as a Fitbit. Two participants described the smartwatch as easy to wear and difficult to lose. Similarly, when talking about a smart speaker, two participants indicated that they had spoken with Siri (a virtual assistant by Apple Inc.) on their smart devices, while other participants simply thought they had no problem interacting with a smart speaker.

For the chair and bed sensor mat, motion sensor, and hand dynamometer, participants showed positive attitudes as one participant said, *“It (the chair and bed sensor mat) looks like something that I almost forget it was there. It’s not something I need to set up or keep track of or restart or anything like that. It’s very easy, a simple device.”* Other comments on these sensors include *“it (the chair and bed sensor mat) seems like it would be very simple.”, “I like this (hand dynamometer) actually, it’s very simple.”, and “it’s (the motion sensor) not anything I have to wear or maintain.”*

Although participants had no problem installing and trying out the technologies, they questioned the technologies’ usefulness based on their health conditions, needs, and added values. Specifically, participants mentioned that they did not need to use the technology to monitor their health when still healthy and active. Participants indicated that the chair and bed sensor mat would not be useful for them because they were still quite active in their daily life.

After stated *“Yeah, I feel comfortable using it.”*, the same participant followed by *saying, “I don’t think I am at a stage where I need to track this stuff (sedentary behavior). I am fairly active. So practically, you know from a practical point of view, I don't see a use currently."*

*"I can’t really see at this point and possibly that in the future."*

*"I mean, It’d be okay to have one (weight scale), I guess. But I don’t see it as being ... unless the person’s got a real problem, you know, with health issues related to obesity."*

*"I have a scale now and I wouldn’t mind having one like this in the future to detect weight loss, you know, especially with cancer with my parents. That was like a big thing, keeping track of the weight loss. They got more frail."*

Limited added value for some technologies was also reported. Some participants gave up using technology such as a smartwatch due to limited perceived contribution to their health. *“It didn’t contribute with anything to my wellbeing. I am still active. I basically know how many steps I take and all that.”*, said one participant. Another participant felt the technological features and perceived health benefits offered by the smartwatch is merely not appealing by stating, *“I first started to investigate this kind of thing earlier, but stopped doing it. Just no attraction to me.”* Although participants stated they had no problem wearing a smartwatch, they just could not find a need for it like a participant said, *“I wouldn’t have any trouble wearing it, but it’s just em, what it’s telling me I don’t have any use for. I don’t need to know.”*

The participants from all three groups voiced several concerns related to the presented technologies. These concerns include:

- Privacy.
- Adherence.
- Appearance.
- Technical concerns.
- Others.

#### Privacy

The standard camera received the most significant pushback. Fourteen participants reported that they did not feel comfortable having the camera installed in their homes and being monitored 24/7. They felt the use of the camera in their homes was intrusive and violating their privacy. Some of the participants stated, *“It doesn’t fit well with me. I like my privacy.”, “I wouldn’t want to feel that somebody was watching my activities.”, “I feel invasive.”, “No, no use for it (standard camera) whatsoever other than security outside, that’d be fine. But inside those there’s no use at all to me.”* and *“I would not like to use it. I will I would consider it an invasion of privacy.”*

On the contrary, all participants were more positive towards a depth camera, which would not reveal a person’s clear image but only its silhouette. Participants felt more appropriate and more acceptable to this approach. Participants stated, *“I think it (depth camera) is an improvement over a previous technology (standard camera) regarding privacy.”, “I would be much more comfortable with this (depth camera), and I wouldn’t mind, I don’t think if it was in the bedroom. You can’t see what I’m wearing, and you can’t see my face; you don’t’ know anything about my body other than the outline. Yeah. I wouldn’t have an issue.”, “It (depth camera) allows for privacy. Yah, on the other hand, the camera (depth camera) provides information in addition to the individual. It may record the person is not dressing properly, and it could be a further clue of deterioration for the clinician. So, I find a balance between privacy and clinicians to assessing deeply than with a regular camera.”* and *“My face is not seen. So, what’s my problem? I don’t have any problem.”*

#### Adherence

The second most mentioned concern is the adherence to technology, especially for body-worn technologies like the smartwatch. Participants noted that people could find it hard always to remember to wear the smartwatch or forget to wear it in urgent situations. One participant stated as a general comment for all technologies, *“So everything that’s automated I would prefer over something that I have to interact.”*

Skepticism about technologies could be another reason for poor adherence, particularly for body-worn technologies. Like one participant said, *“I would like to try, but it’ll probably go the way (giving up) of all the other things that I’ve tried.”* This participant did not think the technologies would work, so he gave up using them. Three more participants also shared that they had stopped using a smartwatch that they used to use because they did not think it would work or be attractive. *“It didn’t contribute to anything.”*, said one participant. *“I first started to investigate this kind of thing earlier, but stopped doing it. Just no attraction to me.”*, said another participant.

#### Appearance

Participants would be more likely to use technology if the technology is aesthetically pleasing. Two participants stated, *“I think that something like that (chair and bed sensor mat) is probably very practical for a senior, especially if they could put a pillow over top of it to make it a little bit less, more cosmetic and less obtrusive.”* and *“But, aesthetically, I think this is I can’t see it (chair and bed sensor mat) getting used in a practical point of view until somebody became so old that they really didn’t care what, like this, to have this sitting in the middle of their living room or something. I think, you know, uh, I don’t see this is something that somebody will use in the long term.”*

#### Technical concerns

The concerns around the technical aspects of the technologies include the data accuracy, operating interface, interaction with the technologies, and power supply. Participants across all three focus groups questioned whether a motion or fridge door sensor could differentiate people living in the same house as people may live with their spouses, children, or roommates. Three participants stated:

*"My grandson comes over, and the door (fridge door) is open every minute. They’re in and out all the time."*

*"I rent a room from a friend of mine who has a Filipino girlfriend and three little Filipino girls. And the fridge is open five hundred times a day. I might open the fridge three times a day. They open it five hundred times a day. So, for me too, it’s not going to detect anything from for me."*

*"It (motion sensor) cannot differentiate. So, if you live with somebody else or have guests over, it would pick up their activity too, so that’s kind of not beneficial."*

The locations for food storage could also affect data accuracy. Participants expressed their concerns as follows:

*"There’s very little stuff in my fridge other than water and ginger ale,” “that’s not the only place you get food is out of the fridge."*

*"That wouldn’t work for nutrition, like if I open the door to get a bottle of water, that’s not nutrition."*

*"You can prepare meals from your fridge. But you could also make a sandwich and a soup and heat it up. You’re still eating, but you are not using your fridge."*

The concern over the operating interface of the technologies was focused on the camera. One participant said she would find it helpful if the technology could visually present her what data would be shared with her clinician.

*"It would really help me a lot if, as part of this presentation, you had a second photo that says this is what the clinician or the person on the end of the viewing can actually see."*

Technologies such as motion sensors, door sensors, or cameras do not require its user to operate; therefore, there were no concerns about the interaction with the technologies. However, participants were concerned about interaction with the smart speaker, which required its user to answer questions verbally and periodically. One participant asked, *“What if I missed it* (a smart speaker asking questions).”

The last concern was about the power supply of the chair and bed sensor. Two participants expressed opposite concerns, with one preferring battery power and another preferring plug-in power supply.

Others

Other concerns include feeling guilty *(“I don’t know if I’d use that, that would make me feel guilty if I use something like that because then I would know how sedentary I am.”)*, cost *(“All of this technology I have an issue with a lot of seniors I know are living on very low incomes and they have like no extra money to spend on even a Fitbit”*).

### B) Conditions for accepting certain technologies

 Participants’ positive attitudes were built on certain conditions for some of the technologies. In other words, participants would become more acceptable to certain technologies if certain conditions were met. These conditions were summarized in Table 4.

An interesting finding is that non-wrist-worn wearable sensors such as a necklace sensor or ankle sensor might be more appropriate for a particular older population to accommodate their special conditions such as dermatitis. One participant who has been a Fitbit user indicated that she had dermatitis and did not like to wear things on her wrist. Instead of wearing a Fitbit watch on her wrist, she uses a Fitbit clip clipped to her clothes to monitor her health. Another participant stated, *“…also something that you could attach to your ankle or something.”* The third participant liked the sensor to be worn as a necklace by saying, *“something like a necklace…you know like wearing a necklace that would detect some frailty things and make it attractive enough that you can just like wear it as a necklace or something like that.”*

Participants believed that people with specific health issues could become more inclined to use technologies to monitor their health. The reason is when the benefits of technology would outweigh the negative aspects (e.g., invasiveness) in the case of severe health risks or medical conditions. People with a high risk of falls, who are currently in rehabilitation, or currently living alone, could be keener and more acceptable to use technologies. They may even consider using invasive devices, such as a standard camera if their health conditions changed.

One participant commented on her opinion about the standard camera as *“It’s probably good for somebody that’s in recovery and being in their bedroom in recovery. And then you know, their physiotherapist comes in, and their occupational therapist comes in, and so you have to stand up five times today. You gotta roll over, sit up, stand up.”* Two participants who indicated that the standard camera was invasive also expressed that this could change with the decline in their health by saying, *“This may change if my situation, my healthy situation, my problem changed dramatically. In that case, the boundary will be moved a little bit.”* and *“Perhaps if I was in a different place in terms of my frailty, I might be more open to it.”*

For the chair and bed sensor mat, one participant suggested that the sensor mat could be useful for people with lower back issues as the mat could remind people to change their postures if they stayed in a sedentary position for too long. *“I think it’s good, you know, having that kind of reminder for some people with probably some lower back issues because it’s you know I was that long lying down, right? You know, they will think, all well that’s I got to change that but if they have if their beds telling the truth or their chair is telling the truth back. They can’t argue.”* Another participant indicated that the mat could be useful for caregivers caring for someone who could not leave the bed.

*"If someone is declining so much that they can’t get out of bed or they have to, then this might be useful for a caregiver to record how many hours somebody is lying in one position and they need to be turned, so they don’t develop bedsores. It could be useful to a caregiver for somebody who’s really declined a lot. So I do see some additional plus possible pluses for this."*

Participants also believed a more appropriate installation location and monitoring time for certain technologies could make a difference in accepting the technologies. Four participants expressed a similar idea that they would become more open to use a standard camera if the camera was only installed at a specific location or a public space in their homes, such as a living room or a dining room. *“I feel invasive unless it (the camera) was only kept to say one certain room. But I wouldn’t feel good about it being in a bedroom or washroom,”* said one participant. Participants were not convinced that the door sensor installed only on the fridge was useful for measuring food intake. However, by adding the door sensors on kitchen cabinet doors where food is stored, participants thought the device could become more practical.

For monitoring time, participants would not object to a standard camera if the camera was turned on only during a specific period of just for frailty assessment at a doctor’s request and switched off outside the requested monitoring period. For example, one participant stated, *“I’m gonna enable this for you between 10 in the morning and 11 o’clock tomorrow so you can watch how I walk. That would make sense. But 24 hours a day constantly. No, I’m not comfortable with it.”*

Lastly, technology with assistive functions could promote its acceptance rate. For example, besides the original function of asking questions for self-report exhaustion and food intake, participants found it would be more favorable if a smart speaker can also remind a person to take medication, go out for a walk or measure the grip strength. Like one participant stated, *“I think there is good use of additional, you know, not only ask somebody eat, but also remind somebody to take their medication.”* Three more participants suggested adding a built-in reminder function such as a timer and an alarm to the chair sensor mat and the hand dynamometer.

*"if you’re in a sedentary job, it’s good to get reminded to get up and go for a walk."*

*"I think you’d have to get in your day timer something to remind you to do it (grip strength test using hand dynamometer)."*

*"To have a built-in alarm of any type to alert a senior that, let’s say 30 minutes or an hour had passed. They should make an effort to stand up…The problem is that people can get too sedentary that can be alarming sometimes to realize how long you’ve been sitting in one spot, you know, without moving, and I’m finding as I get older than I’m more and more getting...You could be watching television for all night."*

**Table 4:** Conditions for accepting certain technologies

Technology	Conditions for Acceptance (if applicable)
Smartwatch	• Wearing locations other than on the wrist
Chair and Bed Sensor	• Aesthetic appearance • Has heating function • Has vibration function
Motion Sensor	
Standard Camera	• Only turned on during a limited period at clinician’s request • Only installed at specific areas in a home

### C) Existing living habits or patterns related to the use of the technologies

Participants also shared their living habits or patterns that could influence the use of the technologies. These habits or patterns were centered around three areas: (1) food intake, (2) physical activities, and (3) weight. We found a diverse pattern ranging from food intake habits that had not changed too much for decades to experiencing a gradual change of eating habits, to a significant change in eating habits. Additionally, some participants reported they could not cook anymore and rely on the microwave oven to heat food only, while others mentioned they have a healthy and balanced food intake. Regarding the physical activities, several participants reported that they *“never sit, never had a sitting job”* or walk a lot because of the nature of their jobs (e.g., a chef, or most clients are downtown where walking might be better than driving). In contrast, other participants reported having more sedentary behavior than they used to have.

*"Yeah, I sit a lot more than I used to."*

Moreover,

an apartment’s small size limits participants’ moving around at home and thus fewer physical activities.

*“I don’t move around too much, haha I just live in an apartment.”, said one participant.*

For weight, participants reported diverse weight loss. Some reported unintentional weight loss, whereas others reported constant weight or even weight gain.

**Table 5:** Existing living habits or patterns as related to frailty criteria and the technologies for assessing frailty

Technology	Frailty Criteria	Related Living Habits or Patterns
Smartwatch	Physical Activity, Immobilization	• Regular indoor bike riding • Much outdoor walking for work
Chair and Bed Sensor	Physical Activity, Immobilization	• Few to no sitting due to work
Motion Sensor	Physical Activity, Immobilization	• Has roommates • Small apartment
Standard Camera	Physical Activity, Immobilization	• Wear shorts at home
Depth Camera	Physical Activity, Immobilization	None
Door Sensor	Life Space	• Open and close door without going out
Hand Dynamometer	Muscle Strength	None
Fridge Door Sensor	Food Intake	• Has roommates
Smart Speaker	Food Intake, Exhaustion	• Never eat too much • Significantly change in eating habits • A gradual change in eating habits • Eat the same always • Eat healthily • Cannot cook • Microwave cooking only
Bathroom Scale	Weight	• Constant weight for years • Unintentional weight loss in the past nine years • Weight gain

### D) Constructive suggestions related to the technologies

Participants have given constructive suggestions on the use of the technologies. Some of these suggestions were about other potential uses of the discussed technologies, although some of these suggestions were not directly related to frailty assessment. Participants suggested that there could be more ways to use a smart speaker in addition to assessing self-report exhaustion. For example, older adults could communicate with the smart speaker to mitigate social isolation. Moreover, the smart speaker used to ask exhaustion-related questions could gain more interest from users if it offers added features such as playing music, notifying lab results remotely, and other helpful features. Participants stated that besides collecting food intake information from the smart speaker, it could also suggest to users what to eat based on the food intake information collected from the user. Furthermore, participants thought the smart speaker could ask questions about users’ movements and physical activity.

Technologies would become more acceptable if they could provide more functionalities while performing the core functions for frailty assessment. Just like what a participant said, *“Like if it (bed and chair sensor mat) gets warm and vibrates, I’d love one.”* Another participant also said, *“If it (door sensor) had an alarm on it like a beeping sound if you were concerned about somebody that’s going out on their own. You know, like your spouse, they’d be getting some cognitive disorientation. Or they’re taking medications, and it’s making them a little wonky, you know, sort of like if they go and they are just going to the store to get milk, and you hear the door open if this is going to have like a buzzer on it and close, so you have: ah they’re going, and you’re like ‘’ you’re on the clock’’ because you know how long it’s gonna take for them to go to the store and get back.”*

The groups frequently mentioned the safety of people with cognitive decline or dementia. Participants could see extra benefits of technologies if technology could provide reminders to individuals with cognitive decline when users forget to turn off a stove, feed themselves, close a door, or leave home for a long time.

Once powered on, the environment embedded technologies such as camera, motion, door, and pressure sensors are designed to operate automatically and continuously at home without much manual operation needed from the users, such as turning on and off every day. The continuous operating technologies at home raised concerns from participants; however, most of the concerns came from the camera mentioned in the previous section. In turn, the participants made suggestions about if the technology can be switched from continuous monitoring to be turned on and off only at the doctor’s request.

**Table 6:** Constructive suggestions related to the technologies for assessing frailty

Technology	Suggestions
Smartwatch	• Reminder for completing a certain amount of daily activity
Chair and Bed Sensor	• More functions including getting warm and vibration
Motion Sensor	• Safety reminder for people with cognitive decline or dementia
Standard Camera	• Use in certain rooms • Can be turned on and off by doctors • Use outside of the home • Use for individuals in recovery and monitor compliance to doctor’s plan
Depth Camera	None
Door Sensor	• Safety reminder for people with cognitive decline or dementia
Hand Dynamometer	• Reminder for use • Strength test for other parts of the body • Bone density test
Fridge Door Sensor	• Reminder for food intake for people with cognitive decline or dementia
Smart Speaker	• Reminder for taking medication • Can be used to mitigate social isolation • Engage older adults and avoid resistance to technology by building in more functions such as playing music
Bathroom Scale	None

## Discussion and Implications

This study identified four areas regarding the use of technologies for assessing frailty at home: (A) general attitude towards using the technology, (B) conditions for accepting certain technologies, (C) existing living habits or patterns related to using the technologies, (D) constructive suggestions related to the technologies. Participants generally have no problem with installing and using the technologies presented except for the standard camera. The factors that could affect technology’s acceptability include past user experience, easy to use and maintain, not easy to lose, lightweight, functionality, and clinician’s request. Privacy is the biggest concern for the rejection of the standard camera. Older adults often avoided or stopped using technology due to privacy and security concerns or violations, which also affect their intentions to purchase and use emerging technologies [31]. This study shows that their attitudes would change if privacy issues are no longer a concern. When technology is designed and developed, technology designers and developers need to protect older adults’ privacy for enhancing the adoption of developed technologies.

The study also found that participants did not see a need to be monitored by technologies as they perceived themselves as still healthy and active. Participants did not have a strong understanding that the purpose of using technologies at home is to help early detect less perceivable health changes that could lead to frailty to prevent or delay frailty. Therefore, it will be important to overcome this barrier (older adults’ feeling that they do not need the technology) for older adults who view themselves as healthy to understand the benefits and embrace adopting these technologies.

The acceptance of the standard camera could change if used under certain conditions. Specifically, older adults could become more acceptable to camera monitoring if the camera is only used in a particular area of a home (i.e., living room) or when a person’s health conditions change dramatically. On the one hand, participants realize that the vision-based technologies (i.e., a camera) have advantages over wearable and other furniture embedded sensors like motion or door sensors for gait and posture analysis and fall detection. When a camera is used under the above conditions, the benefits a person gets would outweigh the privacy they give up; therefore, users could become more open to this approach for in-home monitoring. On the other hand, when a person’s health conditions change dramatically, participants believe that they would care less about privacy and become more motivated to try technologies including the camera, as the person’s primary goal has become maintaining and improving health and safety. For non-intrusive technologies such as motion or door sensors presented in this study, participants were skeptical about specific applications’ effectiveness. For instance, although the fridge door sensor was well accepted to be installed at participants’ homes, participants were uncertain about whether the sensor could accurately measure food intake (nutrition), as the sensor was not capable of identifying who used the fridge and what food was taken. Also, as one fridge door sensor can only be installed on one fridge door and only monitoring the opening and close of that door, food taken activities from other places in a kitchen could not be monitored. Multiple sensors should be installed in different areas containing food to have complete food intake detection than one sensor.

The findings about living habits may inspire technology developers, clinicians, and older adults themselves when they develop or use technologies. For example, the choice of technologies and the interpretation of results could be different for someone who only uses a microwave oven than someone who uses both stove and microwave oven to cook. For someone who “never sit” because of work, the choice of technologies and interpretation of results should consider this factor. Participants share different situations at home for food intakes, such as intaking microwave food only or roommates sharing a kitchen, which could affect the technology’s data accuracy. Therefore, no single monitoring location such as the fridge could accurately detect all food intake or even close. Technology should monitor both fridge and kitchen cabinet containing food. Identification technology such as Radio Frequency Identification (RFID) or vision-based technology should be integrated into a kitchen used by multiple residents to count the fridge’s use by the person of interest only.

Similarly, for monitoring the intensity of physical activity by a motion sensor, the sensor that could not identify a specific person could be installed in private rooms where only the older adult being monitored would use it. It would then be interesting to study whether the activity data collected from only private areas in a home could contribute to frailty assessment. If needed, the identification technologies such as camera or RFID can be added to get more valuable data in public areas. Overall, individualized technology design and development for assessing frailty at home should be considered.

The findings in this study could also influence the form factor (e.g., size, shape, appearance) and functionality of technology for assessing frailty at home. For example, the design of a wearable sensor should consider having various form factors to meet the older population’s different needs based on their personal preferences and health conditions. Moreover, technology with more useful add-on non-clinical functions, such as a warming or vibration function for a bed and chair sensor mat, could improve technology adherence. Thus, it would enable long-term data collection about sedentary behavior to predict frailty better. Similarly, changing how the technology works at home, such as allowing turning on and off technology for a specific period with a clinician’s request instead of continuous monitoring 24/7, could turn the technology more favorable and therefore longer used by its user.

Finally, It was worth noting that the third focus group session was held virtually in Microsoft Teams software due to the COVID-19 pandemic. It was technically feasible to conduct focus group sessions online. The software supported multiple participants to join the same virtual meeting room and hear each other almost in real-time, depending on the network speed. The software also allowed the recording of the meeting. However, there were a few interruptions due to technical issues such as discontinuous voice from individual participants and internet connection loss. The session took a little more time and patience to make sure no topics were missing.

## Conclusions

This study focuses on understanding older adults’ attitudes and perceptions on several technologies that could potentially be used to assess frailty in home settings. Participants generally have positive attitudes towards allowing the technologies to be installed and used at their homes. Some technologies such as cameras were more acceptable if used under certain conditions such as installation locations, monitoring period or with specific populations. However, questions and concerns remain, such as privacy, adherence, appearance, and technical aspects. The study also found that older adults’ living habits or patterns could affect the design and use of technology. Lastly, many valuable suggestions regarding the use of technologies have been made by participants. These suggestions might not be directly related to frailty assessment; however, the technology acceptance and feasibility could be improved if considered.

## Data Availability

The datasets used and analyzed during the current study are available from the corresponding author on reasonable request.

## Abbreviations

RFID: Radio Frequency Identification
IATSL: Intelligent Assistive Technology and Systems lab

## Declarations

All methods were carried out following relevant guidelines and regulations.
